# Gyrate atrophy-like phenotype with normal plasma ornithine and low plasma taurine

**DOI:** 10.3205/oc000131

**Published:** 2020-02-27

**Authors:** Aubhugn T. Labiano, Milagros H. Arroyo

**Affiliations:** 1 Department of Ophthalmology and Visual Sciences, Sentro Oftalmologico Jose Rizal, University of the Philippines Manila – Philippine General Hospital, Manila, Philippines

**Keywords:** gyrate atrophy, chorioretinal atrophy, ornithine, taurine

## Abstract

We present the case of a 39-year-old male with sectoral chorioretinal atrophy similar to that seen in gyrate atrophy (GA) but with a normal plasma ornithine level. Unlike previously reported cases of GA, he had below-normal plasma taurine concentration. Much remains unknown about the pathophysiologic mechanism underlying gyrate-like chorioretinal atrophy. The concomitant occurrence of GA-like phenotype and hypotaurinemia suggests a possible future avenue for research on this matter.

## Introduction

Gyrate atrophy of the choroid and retina (GA) is a rare autosomal recessive metabolic disorder characterized by progressive chorioretinal degeneration, with the atrophic lesions having scalloped or garland-like borders, hence the term “gyrate” [1], [2]. It has been associated with elevated levels of the amino acid ornithine in plasma as well as in aqueous humor, cerebrospinal fluid, and urine [3] . The underlying cause of the hyperornithinemia is a deficiency in the mitochondrial matrix enzyme ornithine aminotransferase (OAT) [4], [5]. The typical clinical history begins in childhood with a decrease in visual acuity associated with night blindness and poor peripheral vision [1], [6]. At the onset of visual symptoms, sharply defined round areas of atrophy are seen in the retinal midperiphery. As these atrophic areas enlarge, coalesce, and spread to the far periphery, the peripapillary area, and even the foveal area, the visual symptoms worsen, the visual fields progressively constrict, and blindness may ensue. 

Occasional reports of cases having fundus findings resembling those typically seen in GA but with normal or mildly elevated plasma ornithine appear in the literature. These patients have been referred to as having normoornithinemic GA, GA-like phenotype, or atypical GA [7], [8], [9], [10], [11].

We present a case with sectoral chorioretinal atrophy similar to that seen in GA but with a normal plasma ornithine level. Our patient also had a low plasma taurine concentration. To our knowledge, this is the first reported case of a gyrate atrophy-like phenotype associated with hypotaurinemia. 

## Case description

A 39-year-old male presented with the complaint of blurred vision, described as haziness, in his left eye of two months’ duration. He acknowledged that he had experienced blurring of vision of both eyes 12 years ago, and that his vision improved and stabilized after using unrecalled topical medications. He reported little or no difficulty with night vision. Aside from occasional tearing, the patient had no other associated ocular symptoms. He had no known comorbid conditions and no systemic complaints. He denied any of his immediate family experiencing similar symptoms. He also denied consanguinity of his parents. 

**Figure 1:**
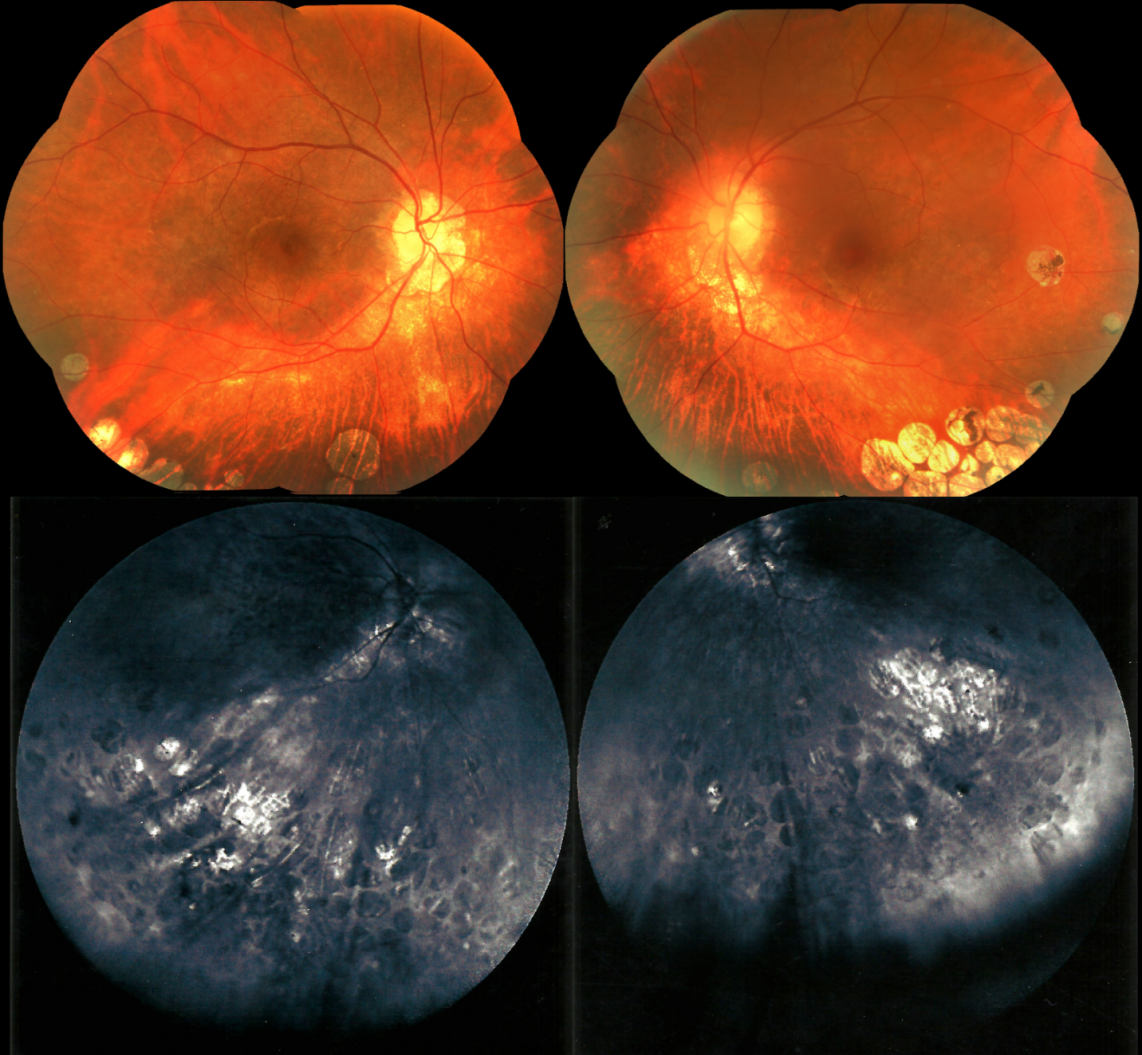
Fundus photographs showing patches of chorioretinal atrophy in the inferior retina of both eyes

Snellen uncorrected visual acuity in the right eye was 20/20, and the manifest refraction was plano. Snellen uncorrected visual acuity in the left eye was 20/100, which was best corrected to 20/20 with –1.25 D sphere =–0.75 D cylinder x90°. Color vision, gross eye examination findings, and intraocular pressures were within normal limits. Slit lamp examination showed a small and thin posterior subcapsular cataract in the left eye. Dilated fundus examination revealed in both eyes roundish gray patches of chorioretinal atrophy, some coalescing to form a gyrate shape, mostly located in the inferior retinal midperiphery and far periphery (Figure 1 (Fig. 1)). No such lesions were seen in the macula. There was also peripapillary atrophy.

**Figure 2:**
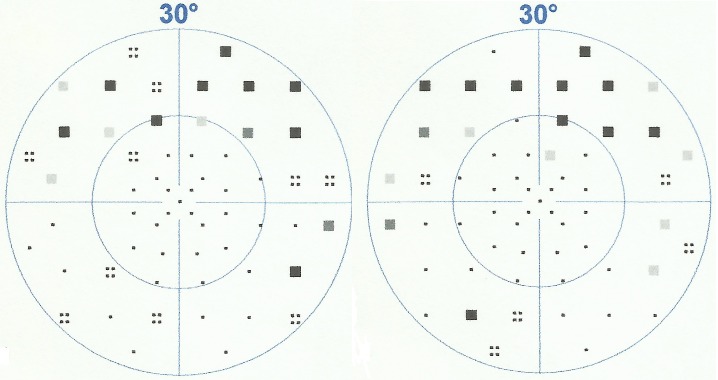
Pattern deviation probability maps showing superior visual field constriction in both eyes

On fluorescein angiography, the patches appeared as hypofluorescent areas with faintly hyperfluorescent borders. Choroidal vessels were prominent within the lesions. Spectral-domain optical coherence tomography (OCT) of the macula revealed normal central foveal thickness in both eyes and perifoveal thinning, which was more pronounced in the left eye than in the right. Horizontal scans of the macula showed no abnormalities. Testing of the central 30 degrees of the visual field with the Octopus 300 perimeter (Haag-Streit AG, Köniz, Switzerland) revealed in each eye a superior arcuate scotoma and enlargement of the blind spot (Figure 2 (Fig. 2)). There was inferior retinal nerve fiber layer (RNFL) thinning in the right eye on OCT of the optic nerve head and RNFL. Full-field electroretinogram (ERG) responses in light- and dark-adapted conditions showed A and B waveforms with amplitudes and implicit times that were generally within the normal range (Table 1 (Tab. 1)). Waveform amplitudes in the left eye were mostly lower than those in the right eye. Multifocal ERG, which is more sensitive than full-field ERG in detecting local retinal dysfunction, was not available in our center and in other centers in the country and thus was not performed. 

Quantitative plasma amino acid analysis done after an overnight fast revealed a normal plasma ornithine level at 61 µmol/L (reference interval: 48–195 µmol/L). Levels of the other amino acids, with the exception of taurine, were within normal limits. Plasma taurine concentration was below normal range at 36 µmol/L (reference interval: 54–210 µmol/L). The amino acid analysis was done using ultrahigh performance liquid chromatography – Waters MassTrak amino acid analysis system. Reference intervals were adapted from the Princess Margaret Hospital for Children, Perth, Australia. Testing for OAT activity and OAT genetic testing were unavailable in our center and in nearby institutions and thus were not done. Nerve conduction studies revealed motor potentials within normal limits and normal F-waves but with reduction of sensory potentials of the right median nerve, indicative of mild carpal tunnel syndrome. Monopolar needle studies on the muscles of the right paracervical and paralumbar areas showed no signs of acute denervation. 

Based on the history, fundus findings, and results of ancillary tests, our patient was diagnosed as having a gyrate-atrophy-like phenotype. The patient was advised spectacle correction of his error of refraction, increased intake of taurine-rich foods, and once- or twice-yearly follow-up for monitoring of the lesions and possible repeat plasma amino acid analysis. Ophthalmologic examination of his parents and siblings was also recommended.

## Discussion

Differential diagnoses for gyrate atrophy include choroideremia and myopic fundus changes [7], [12]. Choroideremia is a rare X-linked recessive disorder. The earliest symptom is night blindness in the first or second decade of life, and there is eventual progressive visual field constriction and visual acuity loss. Early fundus manifestations of choroideremia consist of pigment clumping at the level of the retinal pigment epithelium (RPE) and peripapillary atrophy [13]. In the advanced stage of the disease, the loss of RPE and choriocapillaris can form confluent scalloped areas resembling those seen in the later stages of GA. The well-demarcated round lesions observed in our patient are not characteristic of choroideremia. Patients with high myopia may present with peripheral clusters of atrophic areas or pavingstone degeneration that may resemble the lesions seen in GA [12], [14]. The manifest refraction of our patient did not show a high degree of myopia. Pavingstone degeneration is typically found between the ora serrata and the equator [15]. 

Few reports of atypical GA or GA-like cases appear in the literature. Similar to our patient, they were noted to have the lesions characteristically seen in GA and had normal plasma ornithine levels. Bargum reported on the case of a 67-year-old female patient with late onset of nyctalopia and blurred vision and a mild and protracted clinical course [7]. Kellner et al. described six patients, three from the same family, and three simplex cases, who presented with the typical lesions of gyrate atrophy. The presence of similar signs and symptoms among three members from the same family consisting of a 70-year-old father, the 41-year-old son and the 39-year-old son suggested an autosomal dominant disorder [8]. Saito et al. reported on the case of a 44-year-old woman of consanguineous parentage. Amino acid analysis of her urine revealed increased excretion of proline, hydroxyproline, and glycine [9]. Mishima et al. presented the case of a 3-year-old boy who was diagnosed with the Fukuyama type of congenital muscular dystrophy and high myopia [10]. None of these cases featured a plasma taurine level below the normal range. 

Hypotaurinemia has also not been previously reported in association with the typical gyrate atrophy with hyperornithinemia. Berson studied the plasma amino acid levels of 41 patients with hereditary retinal degenerative diseases, among which five had gyrate atrophy, six relatives of patients diagnosed with GA, and 13 normal subjects. Mean plasma ornithine was found to be significantly elevated among the GA patients and, to a lesser extent, among their relatives. Mean plasma lysine level among GA patients was found to be significantly lower than that among the normal subjects. In all subjects with gyrate atrophy as well as in all other study participants, plasma taurine levels were within normal limits [16].

The pathophysiologic mechanism of the retinal degeneration seen in GA with hyperornithinemia is not yet fully understood. It has been hypothesized that this is due to the toxicity of high ornithine levels to chorioretinal tissue [17], [18]. Decreased levels of Δ^1^-pyrroline-5-carboxylate, proline, or phospocreatine have also been proposed to underlie the chorioretinal degeneration in GA [18], [19], [20]. Much less is known about the pathophysiology of GA-like lesions associated with normal ornithine levels. Kellner et al. posit that there may be a common but still unknown disease process at play in hyperornithinemic and normoornithinemic cases or that diverse conditions may result in the same chorioretinal degeneration [8]. Saito et al. suggest that a deficiency in proline may be relevant in the development of atypical and typical GA [9]. Determining the link between low plasma taurine and GA-like phenotype, if it at all exists, is beyond the scope of this case report. Future research on the possible association of these two findings is suggested.

Taurine or 2-amino-ethanesulfonic acid is a free non-protein sulfur-beta-amino acid. It is a non-essential amino acid that can be synthesized from L-methionine and L-cysteine, although most taurine comes from the diet. It is the most abundant amino acid in the mammalian retina. Of all tissues, the retina has the highest concentration of taurine. The specific function played by taurine in the retina is still unclear. Taurine is considered to be an antioxidant after being found to directly and indirectly neutralize reaction oxygen species. It could play a role in decreasing oxidative stress, which has been implicated in the development of retinal degenerative disorders. It is also considered to be neuroprotective because it acts to regulate the cellular concentration of calcium and could prevent glutamate excitotoxicity [21].

In cats, taurine deficiency has been associated with retinal degeneration, although not in the pattern and location of the lesions seen in gyrate atrophy. The initial fundus finding is a hyperreflective granular zone in the area centralis, which corresponds to the human macula. Microscopically, there is photoreceptor outer segment degeneration in that area. Subsequently, there is progressive loss of the entire photoreceptor population [22]. Children on long-term parenteral nutrition with mean plasma taurine levels less than half that of the control group have been found to have delayed photopic and scotopic b-wave ERG implicit times and some have been observed to have RPE granularity in the posterior pole [23]. Taurine deficiency has been implicated as a cause of the retinal toxicity induced by vigabatrin, which is known to cause visual field constriction [24]. Vigabatrin-treated rats have been found to have photoreceptor damage and retinal ganglion cell loss [25].

## Conclusions

We described the case of a patient with chorioretinal lesions characteristic of gyrate atrophy but with normal plasma ornithine level. Unlike other reported cases of GA, our patient had below-normal plasma taurine concentration. The concomitant occurrence of GA-like phenotype and hypotaurinemia may be more than fortuitous and suggests a future avenue for research on the pathophysiology of gyrate-like chorioretinal atrophy.

## Notes

### Competing interests

The authors declare that they have no competing interests.

## Figures and Tables

**Table 1:**
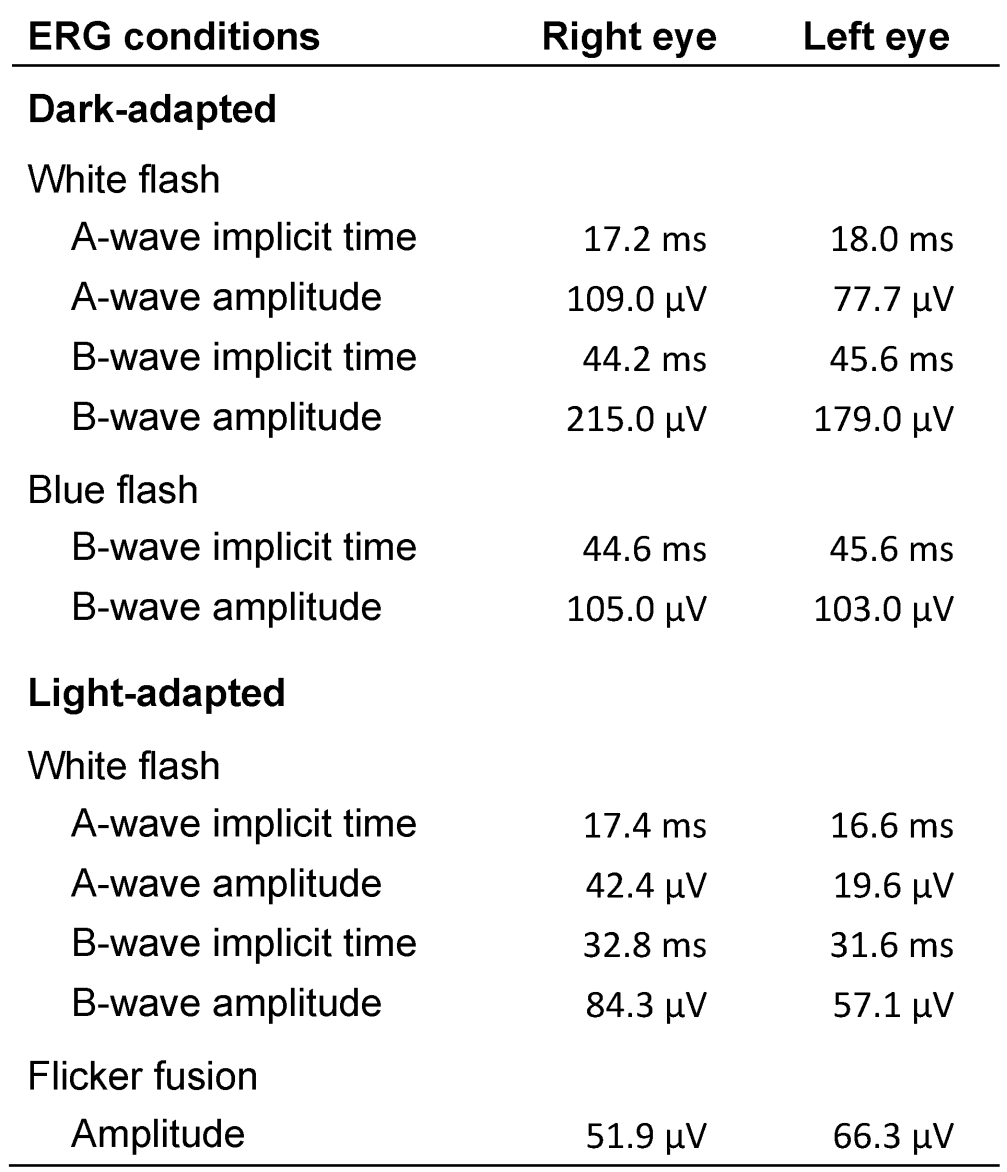
Full-field electroretinogram results

## References

[1] Takki K (1974). Gyrate atrophy of the choroid and retina associated with hyperornithinaemia. Br J Ophthalmol.

[2] Takki K, Simell O (1974). Genetic aspects in gyrate atrophy of the choroid and retina with hyperornithinaemia. Br J Ophthalmol.

[3] Simell O, Takki K (1973). Raised plasma-ornithine and gyrate atrophy of the choroid and retina. Lancet.

[4] Valle D, Kaiser-Kupfer MI, Del Valle LA (1977). Gyrate atrophy of the choroid and retina: deficiency of ornithine aminotransferase in transformed lymphocytes. Proc Natl Acad Sci USA.

[5] Kaiser-Kupfer MI, Valle D, Del Valle LA (1978). A specific enzyme defect in gyrate atrophy. Am J Ophthalmol.

[6] Takki KK, Milton RC (1981). The natural history of gyrate atrophy of the choroid and retina. Ophthalmology.

[7] Bargum R (1986). Differential diagnosis of normoornithinaemic gyrate atrophy of the choroid and retina. Acta Ophthalmol (Copenh).

[8] Kellner U, Weleber RG, Kennaway NG, Fishman GA, Foerster MH (1997). Gyrate atrophy-like phenotype with normal plasma ornithine. Retina (Philadelphia, Pa).

[9] Saito T, Hayasaka S, Yabata K, Omura K, Mizuno K, Tada K (1981). Atypical gyrate atrophy of the choroid and retina and iminoglycinuria. Tohoku J Exp Med.

[10] Mishima H, Hirata H, Ono H, Choshi K, Nishi Y, Fukuda K (1985). A Fukuyama type of congenital muscular dystrophy associated with atypical gyrate atrophy of the choroid and retina. A case report. Acta Ophthalmol (Copenh).

[11] Bhakhri R, Ridder WH (2016). Gyrate Atrophy-Like Phenotype: Normal Plasma Ornithine and Retinal Crystals. Optom Vis Sci.

[12] Arshinoff SA, Leung K, Strube YNJ, Tasman W, Jaeger EA (2006). Gyrate atrophy (Chapter 25). Duane's Ophthalmology.

[13] Khan KN, Islam F, Moore AT, Michaelides M (2016). Clinical and Genetic Features of Choroideremia in Childhood. Ophthalmology.

[14] Karlin DB, Curtin BJ (1976). Peripheral chorioretinal lesions and axial length of the myopic eye. Am J Ophthalmol.

[15] O’Malley P, Allen RA, Straatsma BR, O’Malley CC (1965). Paving-stone degeneration of the retina. Arch Ophthalmol.

[16] Berson EL, Schmidt SY, Rabin AR (1976). Plasma amino-acids in hereditary retinal disease. Ornithine, lysine, and taurine. Br J Ophthalmol.

[17] Kuwabara T, Ishikawa Y, Kaiser-Kupfer MI (1981). Experimental model of gyrate atrophy in animals. Ophthalmology.

[18] Kaiser-Kupfer MI, Valle DL (1987). Clinical, biochemical, and therapeutic aspects of gyrate atrophy. Prog Retin Res.

[19] Saito T, Omura K, Hayasaka S, Nakajima H, Mizuno K, Tada K (1981). Hyperornithinemia with gyrate atrophy of the choroid and retina: a disturbance in de novo formation of proline. Tohoku J Exp Med.

[20] Sipilä I, Simell O, Arjomaa P (1980). Gyrate atrophy of the choroid and retina with hyperornithinemia. Deficient formation of guanidinoacetic acid from arginine. J Clin Invest.

[21] Froger N, Moutsimilli L, Cadetti L, Jammoul F, Wang QP, Fan Y, Gaucher D, Rosolen SG, Neveux N, Cynober L, Sahel JA, Picaud S (2014). Taurine: the comeback of a neutraceutical in the prevention of retinal degenerations. Prog Retin Eye Res.

[22] Hayes KC, Carey RE, Schmidt SY (1975). Retinal degeneration associated with taurine deficiency in the cat. Science.

[23] Geggel HS, Ament ME, Heckenlively JR, Martin DA, Kopple JD (1985). Nutritional requirement for taurine in patients receiving long-term parenteral nutrition. N Engl J Med.

[24] Jammoul F, Wang Q, Nabbout R, Coriat C, Duboc A, Simonutti M, Dubus E, Craft CM, Ye W, Collins SD, Dulac O, Chiron C, Sahel JA, Picaud S (2009). Taurine deficiency is a cause of vigabatrin-induced retinal phototoxicity. Ann Neurol.

[25] Jammoul F, Dégardin J, Pain D, Gondouin P, Simonutti M, Dubus E, Caplette R, Fouquet S, Craft CM, Sahel JA, Picaud S (2010). Taurine deficiency damages photoreceptors and retinal ganglion cells in vigabatrin-treated neonatal rats. Mol Cell Neurosci.

